# A comparative study between two models of active cluster crystals

**DOI:** 10.1038/s41598-019-52420-1

**Published:** 2019-11-13

**Authors:** Lorenzo Caprini, Emilio Hernández-García, Cristóbal López, Umberto Marini Bettolo Marconi

**Affiliations:** 1 Gran Sasso Science Institute (GSSI), Via. F. Crispi 7, 67100 L’Aquila, Italy; 2 IFISC (CSIC-UIB), Instituto de Física Interdisciplinar y Sistemas Complejos, Campus Universitat de les Illes Balears, E-07122 Palma de Mallorca, Spain; 3 Scuola di Scienze e Tecnologie, Università di Camerino - via Madonna delle Carceri, 62032 Camerino, Italy

**Keywords:** Statistical physics, thermodynamics and nonlinear dynamics, Statistical physics, Fluid dynamics

## Abstract

We study a system of active particles with soft repulsive interactions that lead to an active cluster-crystal phase in two dimensions. We use two different modelizations of the active force - Active Brownian particles (ABP) and Ornstein-Uhlenbeck particles (AOUP) - and focus on analogies and differences between them. We study the different phases appearing in the system, in particular, the formation of ordered patterns drifting in space without being altered. We develop an effective description which captures some properties of the stable clusters for both ABP and AOUP. As an additional point, we confine such a system in a large channel, in order to study the interplay between the cluster crystal phase and the well-known accumulation near the walls, a phenomenology typical of active particles. For small activities, we find clusters attached to the walls and deformed, while for large values of the active force they collapse in stripes parallel to the walls.

## Introduction

Active particle models have been introduced to investigate the dynamics of individuals or objects able to convert energy from the environment into directed motion^1–4^. The active systems most studied so far are bacteria^5^, protozoa^6^, spermatozoa^7^, cells, living tissues^8^, actin filaments^9^, active nematics^10^, and the so-called motor-proteins^11^. Recently, active microswimmers have been synthesized in labs^3^. Typical examples are the Janus particles^12^, spherical objects containing two faces of distinct properties, such as hydrophobicity and hydrophilicity. This asymmetry produces a self-propulsion in a given direction. The “activation” of a complex microswimmer could have important applications, for instance in drug delivery: a propulsion mechanism could enhance and direct the transport process with a consequent increase of its efficiency. Alternatively, some colloids could be activated by light or magnetic fields^13^.

A large interest in the polymers community resides in the synthesis and study of star polymers^14^ or dendrimers^15,16^, which have highly branched structures and thus endowed with several interesting characteristics such as globular, void-containing, shapes which make them suitable for the delivery of anticancer drugs and imaging agents. As a consequence of the presence of cavities and channels in their interior, the interaction between different dendrimers or star polymers in solution could be modeled by means of soft-core interactions, which do not prevent the overlap between particles. In particular, it may be described by Generalized Exponential Model (GEM) potentials whose properties have been reviewed by Likos^17,18^. Following this, we adopt a coarse-grained model which replaces a suspension of complex polymers with overlapping spherical particles. Remarkably, under equilibrium conditions, these particles may bind together to form clusters and these, in turn, may organize periodically to form cluster crystals^19–23^. Because of the peculiarities of two-dimensional systems^24^, this would not be a true crystal phase with long-range positional order, but it displays a clear inhomogenous and periodic distribution of particles, much more clustered and ordered than the homogenous state found at high temperature. Such an interesting aggregation phase occurs also in the presence of other soft-core potentials besides the GEM ones, such as the ultra-soft core potentials used for low-temperature bosons^25–27^ and vortices in superconductors^28^.

The self-propulsion is often modeled by means of an effective stochastic force. Perhaps, the simplest model introduced in the literature to describe the behavior of some bacteria populations is represented by the discrete run-and-tumble particle dynamics^29,30^. Recently, suitable descriptions in terms of continuous stochastic processes have received large attention for the possibility of applying well-known tools of statistical mechanics towards the development of the thermodynamic of active microswimmers. In this framework, we mention the active Brownian particles (ABPs)^31,32^ and the active Ornstein-Uhlenbeck particles (AOUPs)^33–36^ models. Regarding the observed phenomenology, there is a strong resemblance between ABP and AOUP, but they also display important differences: (a) the ABP active force has a fixed strength whereas its orientation fluctuates in a diffusive fashion, while in the AOUP there is no such a constraint and the propulsive force fluctuates both in strength and direction; (b) the correlations are of Gaussian type in the AOUP and non-Gaussian in ABP, in spite of the fact that they share the same two-time self-correlation of the active force^37,38^. So far, the practical consequences of these differences have not been completely elucidated. One can say that in the majority of cases the two models display a similar phenomenology, whose characteristics are:

(i) In the absence of external forces some single particle properties such as the diffusion and the mean square displacement have the same form in ABP^31,39,40^ and AOUP^41^.
(ii) Both models undergo the so-called motility induced phase separation^42–45^. While such a topic has been well studied in the ABP case, with steric interactions^46–49^, in the presence of an attractive component of the potential^50,51^ and for more complex interactions^52,53^, the result with AOUP model appeared only in^54^.
(iii) In the two models particles accumulate in the proximity of walls^55,56^. In particular, the wall induces a non-uniform density profile decaying with a characteristic length-scale^57–59^.
(iv) The dynamics in the presence of a convex, radial and non-harmonic potential shows particle accumulation far from the minimum of the potential^12,37,60^ when the activity is large, in such a way that the particle distribution is not Boltzmann-like^61,62^.

The extension of the ABP and AOUP models of activity to particles interacting with soft potentials is considered in the present paper. We analyze how the morphological properties of the system of particles depend on the specific modeling of the active force and if some features of the dynamics are model-independent. What happens if the non-equilibrium forcing is replaced by a colored noise term as in the AOUP model? As an example, it has been found with ABP driving that for some values of the activity parameter the clusters deform into rings with an empty interior^63^, at variance with the equilibrium situation where clusters are compact. We will see here that such an empty-cluster crystal phase does not appear in the AOUP description.

Hereafter, we shall study in detail some properties, such as phase diagram and cluster size, stressing similarities and differences between the AOUP and ABP models. Moreover, motivated by microfluidics applications, we confine the system in large channel to evaluate the long-range influence of the walls on the active cluster-crystal-phase.

After introducing the active ultrasoft model in the ABP and AOUP versions, we present a numerical study, for high enough density, displaying a traveling cluster-crystal aggregation phase. The role of the active force is elucidated, determining at first the size of the cluster and then the occurrence of unstable regions, where clusters shrink and reform. Then, an effective description of the the system is developed, with the aim of describing both the microscopic dynamics of a particle within a cluster and the global dynamics of the pattern. Finally, we confine the system in a large channel to explore the interplay between the cluster-crystal phase and the accumulation near the walls. In the last Section we summarize the results discussing future perspectives in the conclusive section.

## Model

Polymers are often described as large complex structures having many internal degrees of freedom, but in some cases it is not necessary nor possible to take into account their internal properties. For instance, when the resolving power of instruments is low some details of their structure can be disregarded and the polymers can be assimilated to diffusing objects. It is coherent with such an approach to represent the effective interaction among different polymers by a pair-wise potential which depends on the coordinates of their centers of mass. In some cases, such as for dendrimers, this interaction is repulsive and of the soft-core type^17^, basically due to non-diverging potentials which do not prevent the overlap among such complex structures. An appropriate model for the effective interaction is the so-called GEM-*α* potential which reads:

1 $$V(\{{\bf{x}})\})\propto \sum _{i < j}\,\varphi ({{\boldsymbol{x}}}_{ij}),\,\varphi ({{\boldsymbol{x}}}_{ij})=\varepsilon {e}^{-{(|{{\boldsymbol{x}}}_{ij}|/R)}^{\alpha }},$$

being *α* a positive real number, and {|***x***_*ij*_|} are the relative distances between the pairs of particles. We study a system of point particles which self-propel and interact with this potential in two dimensions. Depending on the stiffness of the potential (in particular, *α* > 2 is needed), the passive system (i.e. without the active self-propulsion) shows the occurrence of a peculiar aggregation phase at equilibrium^16,20,22,23^: particles form stable clusters, which arrange into a periodic configuration. In two dimensions the triangular or hexagonal lattice is the only stable pattern, occurring for small enough temperature *T* at a large density. The number of particles, *N*_*c*_, of each cluster depends on the global density and on the typical interaction length, *R*, while the typical inter-cluster distance is determined just by *R*. When the particles are no longer point-like but of small finite size^19,64–66^ similar properties are found, with a new low-temperature phase -the so-called crystal cluster-crystal phase- in which particles inside the clusters also show an ordered structure. The cluster-formation phenomenon despite the repulsive interaction between all the particles can be physically interpreted by considering the force balance between the intra-cluster repulsion (the force felt by particles inside clusters) and the inter-cluster effective interaction, that is, the force exerted by neighboring clusters^19,20^. The increasing of temperature enlarges the typical size of clusters, destroying any structure for *T* sufficiently large.

As mentioned in the introduction, these complex structures could be “activated” by chemical reactions or biological mechanisms taking place inside or on the surface of such a complex microswimmer, so that each individual self-propels in a preferential direction in the same way as a simple rod-like active particle in the absence of any structure. The occurrence of a driving velocity could deform the structure of the polymer, destroying its circular symmetry and altering the shape of the effective interaction given by Eq. (1). But when the speed induced by the self-propulsion is smaller than the typical velocities of the “microscopic” components of the polymer (for instance, the arms of star polymers) then the structural changes of the polymer shape would be negligible and our description in terms of point particles with effective interaction and self-propulsion remains valid. We restrict in the following to this regime.

Self-propulsion can be modeled by means of a force vector, applied on the center of mass of each microswimmer with dynamics independent on the particle position^1,3^. In particular, we consider a two-dimensional system of *N* interacting active particles, whose dynamics is described by over-damped Langevin equations for the positions, **x**_*i*_, of each particle:

2 $$\gamma {\dot{{\bf{x}}}}_{i}={{\boldsymbol{F}}}_{i}+\sqrt{2\gamma T}\,{\eta }_{i}+\gamma {{\bf{f}}}_{i},$$

where ***F***_*i*_ = −▽_*i*_*V* is the total force exerted on the particle *i* by the rest of the particles due to the repulsive potential *V* (as given by Eq. (1)). The term $\sqrt{2\gamma T}{{\boldsymbol{\eta }}}_{i}$ represents the effect of a thermal bath at temperature *T* where active particles are immersed, *η* = (*η*_*x*_*,η*_*y*_) is a two-dimensional Gaussian white noise vector with zero average and correlations 〈*η*_*i*_(*t)η*_*j*_(*t*')〉 = II*δ*_*ij*_*δ*(*t* − *t*'), with II the 2d identity matrix. The constant *γ* is the drag coefficient. The last term, **f**_*i*_, models the self-propulsion mechanism of the microswimmers, and here is where the ABP and AOUP approaches enter into the modelling. In the ABP, **f**_*i*_ is given by a vector of fixed norm: **f**_*i*_ = *U*_0_**nˆ**_*i*_, where **nˆ**_*i*_ = (cos*θ*_*i*_, sin*θ*_*i*_) is a unit vector whose angle, *θ*_*i*_, evolves as a Wiener process

3 $${\dot{\theta }}_{i}=\sqrt{2{D}_{r}}{\xi }_{i}.$$

The constant *D*_*r*_ is the rotational diffusion coefficient, while *ξ*_*i*_ is a Gaussian white noise with zero average and correlations 〈*ξ*_*i*_(*t*)*ξ*_*j*_(*t*')〉 = *δ*_*ij*_*δ*(*t* − *t*'). Instead, in the AOUP model **f**_*i*_ is a noise vector whose components evolve as independent Ornstein-Uhlenbeck processes:

4 $$\tau {\dot{{\bf{f}}}}_{i}=-{{\bf{f}}}_{i}+\sqrt{2{D}_{a}}{{\bf{w}}}_{i},$$

where *τ* is a correlation time, *D*_*a*_ an effective diffusion constant characterizing the active force, and **w**_*i*_ is a Gaussian white noise vector with zero averages and correlations 〈**w**_*i*_(*t*)**w**_*j*_(*t*')〉 = II*δ*_*ij*_*δ*(*t* − *t*'). We point out the relevance of the ratio, *D*_*a*_/*τ*, i.e. the variance of **f**_*i*_, whose square root gives the typical average value of the active force norm.

Despite the differences between ABP and AOUP models, a connection line between them has been already explored in^38^. We remark that AOUP has been originally introduced as a simplification of ABP in order to capture its phenomenology with the aim of making analytical predictions^42^. Subsequently, AOUP was also used to describe the complex behavior of a passive object immersed in a bacterial bath^67–69^. Anyway, AOUP is the simplest Gaussian model which displays at long times the same average and two-time activity-activity correlation function as the ABP. In particular, this correlation in the ABP case has an exponential form, which in two dimensions reads^70^:

5 $$\langle {{\bf{f}}}_{i}(t){{\bf{f}}}_{j}(0)\rangle ={U}_{0}^{2}\langle {\hat{{\bf{n}}}}_{i}(t){\hat{{\bf{n}}}}_{j}(0)\rangle ={\delta }_{ij}{\rm{II}}\frac{{U}_{0}^{2}}{2}{e}^{-{D}_{r}t}.$$

Instead, in the AOUP model, such a correlation is^41^

6 $$\langle {{\bf{f}}}_{i}(t){{\bf{f}}}_{j}(0)\rangle ={\delta }_{ij}{\rm{II}}\frac{{D}_{a}}{\tau }{e}^{-t/\tau }.$$

Setting *τ* = 1/*D*_*r*_ and 2*D*_*a*_/*τ* = *U*_0_^2^ the two-time activity-activity correlation of the AOUP dynamics coincides with Eq. (5)^37^, and the average is 〈**f**_*i*_(*t*)〉 = 0 in both cases.

As commented in the introduction two main differences appear between ABP and AOUP active forces: the fluctuating norm of the AOUP force^38^, and the higher order correlations^71^ which make the ABP force non-Gaussian. We will explore in the following if these differences (and which) are relevant for active cluster crystals.

## Numerical Results

In this Section, we numerically explore the dynamics of Eq. (2) for a suspension of *N* interacting active particles in a two-dimensional box of size *L* with periodic boundary conditions. We implement the Euler-Maruyama algorithm^72^ using both ABP and AOUP active forces, given by Eqs. (3) and (4), respectively. We choose a soft-core potential of the GEM-*α* type with *α* = 3, which displays the cluster-crystal aggregation phase in the passive case (*U*_0_ = 0) at sufficiently low temperature and large enough particle density^20^. Throughout this paper we take *R*/*L* = 10^−1^, *γ* = 1 and *ε* = 1. Since for several active systems of interest the effective diffusion due to the active forces is much larger than the one due to the thermal diffusion, hereafter we fix *T* = (*U*_0_^2^*D*_*r*_/*γ*)10^−4^.

**Figure 1:**
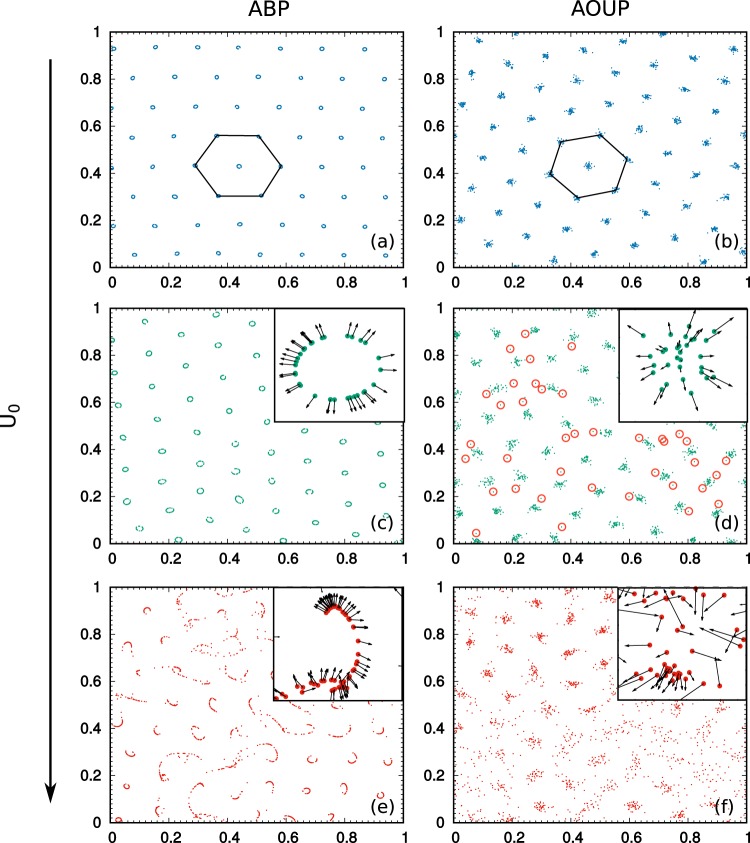
Snapshot of the configurations in the plane *xy* of a system of *N* = 2 × 10^3^ particles interacting with the GEM-3 potential for both ABP (left column) and AOUP (right column) active forces. Panels (a) and (b) are obtained with *U*_0_ = 2.75, panels (c) and (d) with *U*_0_ = 3.75 and panels (e) and (f) with *U*_0_ = 4.5. Other parameters: *γ* = 1, *D*_*r*_ = 1, *L* = 1, *R* = 10^−1^, *ε* = 1, *T* = 10^−4^*U*_0_^2^/*D*_*r*_*γ*. In the AOUP case the parameters used are *D*_*r*_ = 1/*τ* and *U*_0_^2^ = 2*D*_*a*_/*τ*. Black lines in panels (a) and (b) are eye-guides to show the hexagonal pattern. Graphs (c), (d), (e) and (f) display an inset around a small square region around a cluster. In the insets, the black arrows have a length proportional to the active force on each particle. In panel (d), isolated particles not belonging to clusters are highlighted by red circles.

In Fig. 1 we show long-time patterns (*t*/*τ* ≈ 10^2^) for different values of *U*_0_ for ABP (left column) and AOUP (right column). At such a low temperature, both ABP and AOUP form clusters arranged into a hexagonal pattern (see panels (a), (b), (c) and (d)). This scenario resembles the one of passive particles (*U*_0_ = 0)^19,20^.

**Figure 2:**
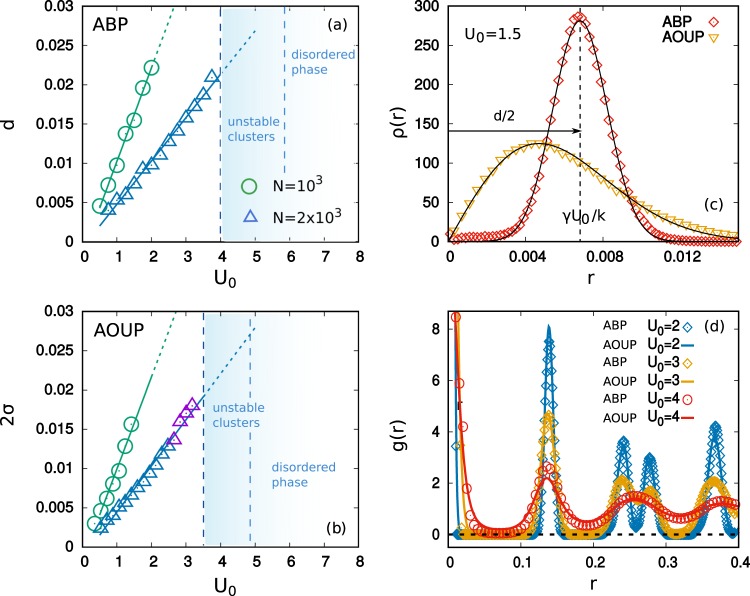
Cluster size as a function of *U*_0_ for two different values of the density: green and blue for *N* = 10^3^, 2 × 10^3^, respectively, at fixed *L*. Other parameters as in Fig. 1. Continuous lines are obtained by linear numerical fits. In the ABP case (Panel (a)) we plot the diameter, *d*, of the ring cluster, while the AOUP case (Panel (b)) we show 2*σ*. In the AOUP case the label *U*_0_ is a short-cut for $\sqrt{2{D}_{a}/\tau }$. The appearance of configurations which exchange particles among clusters is indicated by violet symbols. Blue shades denote the occurrence of unstable clusters in the case *N* = 2 × 10^3^, while the same analysis for *N* = 10^3^ is not shown for the sake of clarity. The left vertical dashed line indicates the smallest value of the active force which displays the instability of at least one cluster in the steady state. The right vertical dashed line shows the beginning of the disordered phase. In panel (c) we show the radial probability density, *ρ*(*r*). Red diamonds refer to ABP, yellow triangles to AOUP. Black lines are the result of a numerical fit. In panel (d) we plot the pair correlation function, *g*(*r*), for different values of the active force both for ABP and AOUP dynamics, as shown in the legend.

Notwithstanding that for low values of *U*_0_ the active force does not change the static macroscopic properties of the pattern, i.e. the hexagonal cluster arrangement, strong differences appear at the dynamical level. While clusters occupy stable equilibrium positions in the passive case (*U*_0_ = 0), without displaying any macroscopic motion, this situation changes in the active case (*U*_0_ > 0). The active forces produce a macroscopic coherent motion of the pattern, which maintains the hexagonal cluster-crystal phase. Clusters, stuck in the hexagonal pattern, drift persistently before changing direction after a time which grows as 1/*D*_*r*_. In movie 1 of the Supplementary Information, we compare the time evolution of systems with *N* = 2 × 10^3^ particles for ABP and AOUP. Despite the total active force acting on each cluster is directed randomly in space (green arrows in movie 1), clusters move coherently in one direction maintaining the hexagonal arrangement. This global drift follows the average active force of the whole system (black arrow in movie 1). The decreasing of *D*_*r*_ enlarges the persistence of the pattern dynamics, for both cases, without producing any significant change on the particles configurations. Traveling crystals^73^ occur also for highly packed suspensions of self-propelled particles interacting by hard-core potentials, i.e. interactions diverging at *r* = 0 in such a way that a finite size is attributed to the particles. Experimental evidence of this effect has been recently studied by means of a suspension of micro-disks subjected to vertical vibrations^74^. The use of density functional theory predicts such a phenomenology and, in particular, the transition to rhombic, quadratic, and lamellar patterns as the active force is increased^73,75^. Such transitions do not occur in our system, where the only stable pattern is the hexagonal one. This statement is confirmed by movie 1 in Supplementary Information and by the study of the pair correlation, $g(r)=\sum _{i\ne 0}\,\delta (x-{x}_{i}){L}^{2}/N$, where a target particle is at the origin, the sum runs over the other particles, and the brackets indicate a circular average over positions **x** with the same modulus |**x**| = *r*. In Fig. 2d), we compare the *g*(*r*) for several values of the active force both for ABP and AOUP dynamics, revealing the occurrence of the typical peaks of a hexagonal pattern, indicating the presence of first, second, third, etc. neighbors at distances 1, $\sqrt{3}$, 2, $\sqrt{7}$, ... times the basic periodicity of the pattern. This periodicity does not change with *U*_0_ and remains at the value determined by this potential in the passive case^19,20^, namely 1.4*R* ≈ 0.14. The increasing of *U*_0_ leads to wider and lower peaks towards the occurrence of the liquid shape of the *g*(*r*). The presence of the initial peak near *r* ~ 0 is due to the *N*_*c*_ particles belonging to each cluster. We remark that the *g*(*r*) remains essentially unchanged when computed at different times during the motion (this is not shown in the figure).

The specific modeling of the active force influences the structure of a single cluster as shown in the insets of Fig. 1, panels (c), (d), for ABP and AOUP, respectively. In particular, ABP’s arrange in ring-like clusters while AOUP’s form Gaussian-like clusters as in the passive case (*U*_0_ = 0). In Fig. 2c) the radial probability distribution, *ρ*(*r*), of a single cluster is numerically measured. *ρ*(*r*)*dr* is the fraction of particles of a cluster which are at a distance between *r* and *r* + *dr* from its center. In particular, *ρ*(*r*) = 2*πrp*(**x**), where *p*(**x**) is the single-particle probability density for a specific particle to be at **x**, which turns out to depend only on the norm *r* = |**x**|.

In the AOUP case, *p*(**x**) is fitted by a Gaussian centered at **x** = 0, while in ABP case *p*(**x**) is close to a Gaussian in *r* = |**x**| centered at a typical radius larger than zero, and displays a smaller variance. For both ABP and AOUP the angular location of the particles in the clusters, i.e. the angular coordinates *ϕ*_*i*_ computed for each particle *i* with respect to the center of its cluster, is approximatively equal to the orientational angle of the active force, *θ*_*i*_. This feature is illustrated in the insets of Fig. 1c,d, where black arrows represent the vectors of the active forces for each particle, which point radially with respect to the center of the cluster. Despite the apparent analogies between the clusters obtained with the AOUP model and the passive systems^20^ (both are non-empty), we note the difference between these two cases: in the large persistence regime, *D*_*r*_ ≪ *γ* an AOUP particle is stuck to its radial position with respect to the center of its cluster only for a time ~*τ*. After this correlation time, the active force is consistently modified and, as a consequence, a large variation in the radial coordinate of the particle occurs. In fact, the non-existence of ring-like clusters in the AOUP is due to the fluctuating norm of the active force at variance with the ABP where it remains constant. This fact is confirmed by simulations (not shown) where a force with the angular time-dependence of the AOUP but a fixed norm is used in analogy with^38^. In this case, empty clusters are obtained. Also, for some particular initial conditions in Eq. (4), ring-like clusters are observed for the standard AOUP, but only as a short-lived (for a time of the order of *D*_*r*_^−1^) transient state.

In Fig. 2 we study the size of a single cluster, i.e. the average diameter of the ring in the ABP case (Panel (a)) or twice its standard deviation in the AOUP case (Panel (b)), as a function of *U*_0_ ($=\sqrt{2{D}_{a}/\tau }$ in the AOUP case). In particular, linear scaling of the standard deviation with *U*_0_ emerges for both types of active drivings and its slope depends on the mean number of particles in each cluster *N*_*c*_: larger values of *N*_*c*_ produce larger confinements. Such a feature is due to the stronger repulsion from the neighboring clusters, which is more important than the intracluster repulsion, and needs the contribution from the active forces to get balanced at a particular ring radius^63^. We remark that such size does not depend on the persistence time of the active force, *τ*. A second difference between the two models emerges at large values of *U*_0_ (or of $\sqrt{2{D}_{a}/\tau }$). As shown by the comparison between Panels (c) and (d) of Fig. 1, at some values of *U*_0_ the AOUP reveals a different phase which does not occur in the ABP model: In panel (d) we observe that some particles, which are marked by red circles, leave their clusters, eventually joining other ones, even if the cluster-crystal phase is preserved. Quantitatively, this particle exchange is a rare event and does not affect the cluster-size as shown in Fig. 2. The number of particles which migrate from a cluster to another, without destroying the stable hexagonal pattern, grows with increasing $\sqrt{2{D}_{a}/\tau }$ in the AOUP but is always a very small fraction of *N*_*c*_. For the ABP case, the exchange of particles could eventually occur only for larger values of *T* as found in^63^, but is always independent of *U*_0_.

A further increase of *U*_0_ leads for both models to the instability of some clusters. In such cases, clusters disappear and immediately reform. The number of unstable clusters increases as *U*_0_ grows, without destroying the global order of the pattern. While in the AOUP case clusters simply blow up, in the ABP case we can clearly see the deformation of the ring-like aggregates into lines after which the cluster can reform. Then, the growth of *U*_0_ produces the formation of continuous flows of particles while other groups of particles arrange in the hexagonal pattern (Fig. 1c,f). In other words, we observe the coexistence of the cluster-crystal phase with the disordered phase. Finally, the formation of any stable structure for both models is prevented by a further increase of *U*_0_, a regime which is not under investigation in the present manuscript.

The comparison between ABP and AOUP shows the weaker stability of the cluster-crystal phase in the latter, due to the particle-exchange mechanism which does not occur in the ABP case. In addition, clusters start to break down for AOUP at smaller values of *U*_0_ as shown in Fig. 2 (for *N* = 2 × 10^3^). The disordered phase in ABP appears for larger values of *U*_0_ than for AOUP. We remark that in both cases the active force plays a role which resembles the one of an effective temperature since its increasing induces a transition towards the disordered phase. In any case, the role of the active force reveals other interesting features, like the coherent motion of the pattern and the shape of a single cluster, which cannot be understood in terms of an effective temperature approach.

## Effective Description

In this Section, we elaborate an effective description of the dynamics of the system in the cluster-crystal phase by separating the dynamics of the single particle from the dynamics of the clusters. This has already been done for equilibrium hard-core passive repulsive particles in^19^ and is based on the fact that the typical distance between clusters remains approximately constant. As commented before, such assumption holds also in the presence of the active force (see the movie in Supplementary Information). These observations allow us to separate the effective dynamics of the single particle from the effective dynamics of the clusters. In particular, the effective equations for the *i*-th particle in the *j*-th cluster reads

7 $$\gamma {\dot{{\bf{x}}}}_{i}^{(j)}={{\boldsymbol{F}}}_{eff}({{\bf{x}}}_{i}^{(j)}-{{\bf{R}}}^{(j)})+\gamma {{\bf{f}}}_{i}^{(j)}+\sqrt{2\gamma T}{{\boldsymbol{\eta }}}_{i}^{(j)},$$

where **F**_*eff*_ is the effective confining force due to the neighboring clusters^63^. The mean cluster positions, **R**^(*j*)^, are located on a hexagonal structure as in the purely Brownian case (*U*_0_ = 0) since the inter-cluster distance does not change significantly by the presence of the active force. The complex dynamics of interacting microswimmers is approximated by a set of independent particles in the presence of a grid of confining potential wells. The validity of this approximation has been discussed in^19,20,23^ in the passive case, and basically follows by a Taylor expansion of the GEM-*α* potential truncated at the second order, since the inter-cluster distance is always larger than the typical cluster-size in the cluster crystal phase. Within this approximation, particles belonging to the same cluster are treated as independent and only experience the effective force generated by the particles in the neighboring clusters^63^, which is described by the linear shape, **F**_*eff*_(**x**) ≈ −*k*(**x** − **R**^(*j*)^). We stress that the same equations are obtained if using interaction potentials different from GEM, the only difference being the particular value of the spring constant *k*. At variance with the equilibrium case where the pattern does not move and each cluster fluctuates around its equilibrium position, in the presence of the self-propulsion the positions **R**^(*j*)^ change. However, since particle relative distances remain constant within the cluster we shall neglect this movement and only consider the particle dynamics inside each cluster and study a system of independent particles confined in a harmonic well in the presence of the self-propulsion. Taking the center of the cluster (**R**^(*j*)^ = **0**) as the origin of coordinates, the effective particle dynamics reads

8 $$\gamma \dot{{\bf{x}}}=-k{\bf{x}}+\sqrt{2\gamma T}\,\eta +\gamma {\bf{f}},$$

where we dropped the indices *i* and *j* for the sake of simplicity.

In the AOUP case Eq. (8) is linear, so that we can solve its associated Fokker-Planck equation for the steady state joint probability distribution function, *f*(**x**,**f**), which reads^57^:

9 $$f({\bf{x}},{\bf{f}})={\mathscr c}\,\exp (-\frac{k}{2}\frac{{|{\bf{x}}|}^{2}}{{D}_{a}\gamma }\frac{{D}_{a}\Gamma }{{D}_{a}+\Gamma T/\gamma })\exp (-\frac{\tau \Gamma }{2{D}_{a}}{|{\bf{f}}-\frac{k}{\gamma }\frac{\Gamma {D}_{a}}{{D}_{a}+T/\gamma }{\bf{x}}|}^{2}),$$

where ${\mathscr c}$ is a normalization and Γ = 1 + *kτ*/*γ* is a numerical factor which depends on *kτ*/*γ* ≫ 1, i.e. the ratio between the correlation time of the activity, *τ*, and the relaxation time, *γ*/*k*, due to the harmonic potential. The result is a multivariate Gaussian distribution with non-zero correlations between each component of **x** and **f**. In other words, a non-zero conditioned first moment of the spatial distribution appears, so that 〈**x**〉 ∝ **f**, meaning that particles prefer to spend their life far from the minimum of the potential in a fixed position determined by the value of **f**. Since the active force is an Ornstein-Uhlenbeck process, **f** can explore large values depending on its variance, *D*_*a*_/*τ*, even if the most probable values remains **f** = 0. Instead, its persistence time, *τ*, rules how long the particle remains close to the particular position determined by the value of **f**.

Integrating out the active force in Eq. (9), we can easily find the probability density for the position of a given particle, *p*(**x**), which reads:

10 $$p({\bf{x}})={\mathscr{N}}{e}^{-\frac{k({x}^{2}+{y}^{2})}{2\gamma }\frac{\Gamma }{{D}_{a}+\Gamma T/\gamma }},$$

where ${\mathscr{N}}$ is a normalization factor. Formula (10) implies that the clusters have a Gaussian shape with 〈*x*〉 = 〈*y*〉 = 0 and defines an effective temperature of the system^33^:

11 $${T}_{e}=k\langle {x}^{2}\rangle =k\langle {y}^{2}\rangle ={D}_{a}\gamma (\frac{T}{\gamma {D}_{a}}+\frac{1}{1+k\tau /\gamma })\approx \frac{{D}_{a}\gamma }{1+k\tau /\gamma }.$$

The last approximation holds if *T* ≪ *γD*_*a*_, the regime considered in this manuscript. Note that the result for *T*_*e*_ is in agreement with the scaling with *τ* recently observed in a dense suspension of active particles with hard-core repulsive interaction, specifically Lennard Jones potentials^76,77^. We also note that Eq. (11) approaches *T* + *γD*_*a*_, in the equilibrium limit, *τ* → 0, i.e. the effective temperature due to the joint effect of self-propulsion and thermal noise for a free microswimmer^31,78,79^. The Gaussianity of the density agrees with the shape of the clusters obtained in Fig. 2c). Moreover, the standard deviation of the above distribution, obtained as the square root of Eq. (11), confirms the linear cluster-size scaling with $\sqrt{2{D}_{a}/\tau }={U}_{0}$, numerically measured in Fig. 2(b). We note that the authors of ^37^ showed that significant anharmonicity of the trap (for instance **F**_*eff*_ = −∇*U*(**x**), with *U*(**x**) ∝ |**x**|^2*n*^ and *n* ≥ 2) would lead, under AOUP dynamics, to a “delocalization” phenomenon, in which particles accumulate far from the minimum of the potential, displaying a non-Boltzmann distribution^54,61,80,81^. The absence of this phenomenon here confirms that the effective trap potential induced by the neighboring clusters is harmonic to a good approximation (see also^63^).

**Figure 3:**
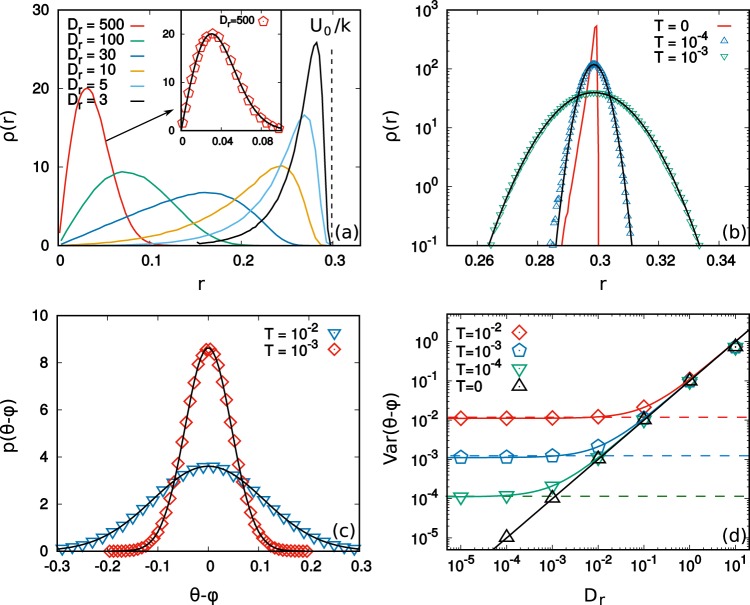
A single ABP particle in a harmonic trap. Panel (a): radial probability density *ρ*(*r*) for different values of *D*_*r*_. The inset shows a comparison between the Gaussian approximation with some effective temperature, Eq. (11), and numerical data for *D*_*r*_ = 500. Panel (b): *ρ*(*r*) for *D*_*r*_ = 0.3 for three values of *T*: colored symbols from numerical simulations, black lines from Eq. (12). The red line is for *T* = 0. Panel (c): angular probability distribution functions, *g*(*θ* − *ϕ*), for two different values of *T* (colored points) compared with Eq. (13) (black lines). Panel (d): Variances of *g*(*θ* − *ϕ*) vs *D*_*r*_ for different values of *T*. Points are obtained by simulations while continuum lines correspond to the variances predicted by the distribution (Eq. 12). The dashed lines indicate the limiting variances obtained for *D*_*r*_ → 0. Other parameters: *k* = 10 and *U*_0_ = 3.

In the case of the ABP active force, in this effective dynamics description in which interparticle forces are replaced by an external confining potential, the solution of the single-particle Fokker-Planck equation is not an easy task even under the simple harmonic potential^60,62,82,83^. Recently, such a system has been studied in the presence of hydrodynamic and steric interactions^84^ by means of the density functional theory^85^. In Fig. 3(a), we numerically study the radial density, *ρ*(*r*) = 2*πrp*(**x**), associated to Eq. (8) with ABP self-propulsion. We identify two regimes: i) when *D*_*r*_ ≫ *k*/*γ*, the activity plays the role of effective temperature and the particle position density *p*(**x**), has a Gaussian form as in the case of AOUP and passive suspensions (see the inset of Fig. 3(a)). In this regime, the increasing of *D*_*r*_ changes only the effective temperature, *γU*_0_^2^/2*D*_*r*_ + *T* (which is equivalent to *γD*_*a*_ + *T* discussed below Eq. (11)), which determines the variance of the distribution. It is straightforward to check that for large *D*_*r*_ the variances of the ABP and AOUP positions coincide. (ii) When ${D}_{r}\lesssim \gamma /k$, particles arrange on a circular crown and the region near the minimum of the harmonic potential becomes empty as in the clusters in Fig. 1. The decrease of *D*_*r*_ enhances the accumulation of particles in the proximity of r^*^ ≈ *γU*_0_/*k* (shown in Fig. 3(a)), where r^*^ corresponds to the radius at which active and the confining harmonic force balance. For *T* = 0 (or *T* small enough with respect to *γU*_0_^2^/*D*_*r*_), the distribution is strongly non-Gaussian and can be approximated by a Dirac *δ*-function centered at *r* − r^*^ in the limit *D*_*r*_ → 0. At *T* = 0 the asymmetry of *ρ*(*r*) is quite evident (see Fig. 3b)), since as the norm of the active force is fixed at *U*_0_, particles cannot explore regions with *r* > r^*^. When ${D}_{r}\lesssim \gamma /k$, particles arrange on the circular crown in such a way that their orientational angles, *θ*, can be approximated by *θ* ≈ *ϕ* = arctan(*y*/*x*), i.e. their angular coordinate with respect to the minimum of the potential. In this case, the probability distribution, *g*(*θ* − *ϕ*), is a Gaussian which becomes narrower as *D*_*r*_ decreases (Fig. 3c)). In Fig. 3(d) we study the variance of *g*(*θ* − *ϕ*) vs *D*_*r*_ for several values of *T*, showing a linear scaling (black triangles).

The effect of *T* is shown in Fig. 3b,c,d. On one hand, the larger thermal fluctuations symmetrize the shape of *ρ*(*r*) leading the system to explore the region *r* > r^*^, otherwise inaccessible. In this regime, *p*(**x**) is well-approximated by a Gaussian concentrated on a ring of radius |**x**| ≈ r^*^, so that the radial density is:

12 $$\rho (r)\approx {\mathscr{N}}r\,\exp (-\frac{k}{2T}{(r-{r}^{\ast })}^{2}),$$

as confirmed in Fig. 3(b). On the other hand, even if *T* does not change the Gaussian shape of *g*(*θ* − *ϕ*), its value can determine the variance of the distribution. For large *D*_*r*_ the variance is independent of the value of *T* (Fig. 3d)). A decrease of *D*_*r*_ determines a deviation from the linear behavior until a *T*-dependent plateau is reached. The shape of *g*(*θ* − *ϕ*) is approximatively described by a Gaussian distribution:

13 $$g(\theta -\varphi )\approx {\mathscr{N}}\,\exp (\frac{{r}^{\ast }}{2}\frac{\gamma {U}_{0}}{T+\gamma {D}_{r}{({r}^{\ast })}^{2}}{(\varphi -\theta )}^{2}),$$

whose variance excellently agrees with numerical simulations as shown in Fig. 3(d). In the Supplementary Information we show an analytical argument, which comes from the analysis of the Fokker-Planck equation, to derive Eqs. (12) and (13).

The study of the distribution of particles inside each cluster, within the present approximation of independent particles in a confining potential allows to further understand the origin of some differences between the AOUP and ABP active forces, occurring at large *U*_0_. We already showed that the constancy of the norm *U*_0_ makes the difference. Another way to see this is by noting that in the AOUP case the fluctuations in the positions are ruled by *T*_*e*_/*k* given by Eq. (11), which scales as ~*γ*^2^*D*_*a*_/*τk*^2^ for large *τ*. In this case, we can find a particle at *r* > r^*^ with a finite probability, which is controlled by the strength of the active force in the regime of very small *T*. A large value of *D*_*a*_/*τ* increases the diameter of the cluster and the probability of finding a particle far away from its most probable value. On the other hand, in the ABP case fluctuations are mostly ruled by *T*, while *γU*_0_/*k* only determines the maximal cluster radius, without increasing the particle positions fluctuations. Only fluctuations induced by *T* could lead a particle to explore radial distances larger than *γU*_0_/*k* from the center of its cluster. This difference explains why in the regime of negligible *T*, ABP does not display the particle-exchange phase, at variance with the AOUP model. This is also the main reason for which the AOUP active force has a region of cluster instability for smaller values of *U*_0_ ($\sqrt{2{D}_{a}/\tau }$). This description directly agrees with the radial distribution measured in panel Fig. 2c), where, despite the same value of *U*_0_, the probability of finding an AOUP-particle for *r* ≳ r^*^ is consistently larger than the ABP counterpart. The particle-exchange phase could occur only if a “small” fraction of particles has an active force large enough to overcome the effective barrier induced by the neighboring clusters, which for *T* → 0 can be provided only by the norm fluctuations of **f**, a mechanism which is absent in the ABP model with *T* = 0. We remark that “small” has to be considered in relation to the average number of particles inside each cluster^86^.

## Active Cluster Crystal in a Channel

In this Section, we return to the original system of *N* active particles interacting through a repulsive GEM-*α* potential, and consider its behavior in a long channel. The aim is studying the interplay between cluster-crystal active aggregation and geometrical confinement.

The cluster crystal phase obtained with soft-core interactions and in the presence of a confining mechanism has been already studied in^27^ in the passive case. The hexagonal pattern is stable if the cross-section of the channel is larger than the typical interaction length of the soft-core potential, *L*/*R* ≫ 1, a regime which will be considered hereafter.

In addition, it is well-known that active particles, also in the non-interacting case, manifest the tendency to accumulate near the walls^87,88^, in the regime of large persistence. Both for AOUP and ABP active forces, a microswimmer maintains its direction roughly during the active force correlation time (1/*D*_*r*_ or *τ*). This persistence induces a profile in the space-density of the system, producing an anomalous maximum in front of each wall higher than the typical bulk density^55,56,89–91^. We, now, evaluate the relation between the cluster crystal phase and the wall-accumulation.

We consider a channel of infinite length in the *y*-direction, and width 2*L* in the transverse *x*-direction and model two parallel walls by means of external repulsive potentials at positions *x* = ±*L*. For the sake of simplicity, we choose a truncated harmonic wall-shape in such a way that the force exerted on the particle, **F**_*w*_, is linear and directed along the direction of the unit vector **xˆ**. In detail:

14 $$\begin{array}{l}{{\bf{F}}}_{w}^{l,r}=-k(x\pm L)\theta (x\pm L)\hat{{\bf{x}}}\,,\end{array}$$

where the indices *l* and *r* refer to the left and the right walls, respectively. *θ*(*x*) is the Heaviside function and the constant *k* represents the strength of the harmonic potential. Along the *y*-direction we consider periodic boundary conditions to mimic the infinite size of the channel. In this way, the equations of motion read:

15 $$\gamma {\dot{{\bf{x}}}}_{i}={{\bf{F}}}_{i}+\sqrt{2\gamma T}\,{\eta }_{i}+\gamma {{\bf{f}}}_{i}+{{\bf{F}}}_{w}^{l}+{{\bf{F}}}_{w}^{r}.$$

It is well-known that active particles show a peculiar behavior in confined geometries^92^. When the active force is strong and persistent the microswimmers accumulate at the walls, so that their stationary probability distribution displays anomalous peaks in the proximity of the walls, at *x* ≈ ±*L*. The main reason is related to the time-persistence of their motion, which keeps the direction of the active forces roughly for a time ~1/*D*_*r*_, depending on the model employed^93^. The number of particles accumulating at the walls with respect to the particles in the bulk is controlled by the persistence length of the active motion, ${\lambda }_{a}={U}_{0}/{D}_{r}\propto \sqrt{{D}_{a}\tau }$. When *λ*_*a*_ ≪ *L* the majority of the particles moves freely in the bulk, while the number of particles at the wall become comparable with the bulk-particles in the opposite regime^86^. In such a situation, the role of *T* has important consequences: the accumulation is reduced and a bulk profile of the density occurs even far from the walls, both for ABP^58,59^ and AOUP^57^ active forces. Such a long-range effect of the wall has not a Brownian counterpart and increases with the ratio *T*/*γD*_*a*_.

**Figure 4:**
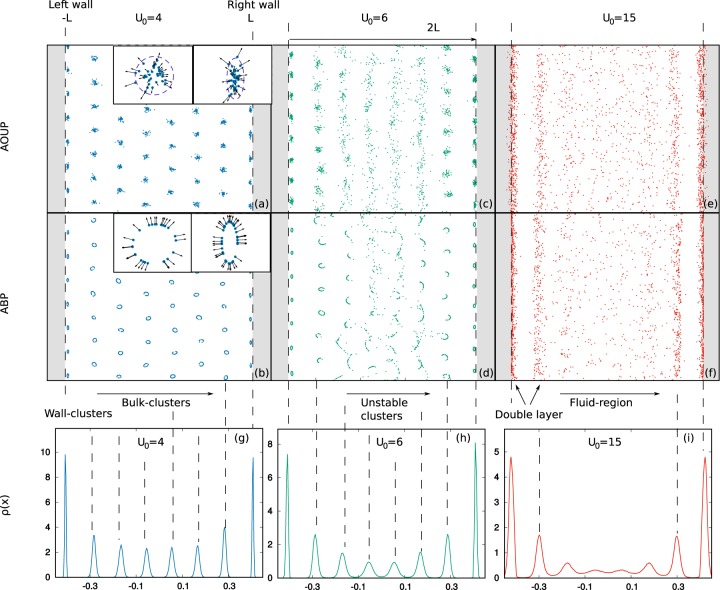
Panels (a–f): Long-time snapshot for *N* = 2 × 10^3^ particles confined by walls at positions *x* = ±*L*. Panels (a), (c), (e) are obtained with the AOUP model while panels (b), (d), (f) with the ABP one. From the left to the right we increase *U*_0_ = 4, 6, 15 ($\sqrt{2{D}_{a}/\tau }$). Grey regions are forbidden due to the presence of the walls. Panels (g–i): spatial density for the AOUP case (similar for ABP -not shown-) along the transversal direction of the channel, in correspondence of the snapshots. Black dashed lines in correspondence of the peaks are guides to the eye. Other parameters are: *ε* = 1, *R* = 10^−1^, *L* = 1, *k* = 10 and *γ* = *D*_*r*_ = 1.

In Fig. 4, we display configurations obtained for three different values of *U*_0_ keeping fixed *D*_*r*_. At small *U*_0_, the cluster-crystal phase occurs and, for both ABP and AOUP, aligns along the wall direction, as shown in panels a), b), c) and d). Interestingly, we find two symmetric narrow stripes of clusters attached to the walls whose shapes are strongly deformed with respect to the bulk-clusters (see the insets in panels a) and b)): in the ABP case, we observe ellipsoidal-like clusters instead of the ring-like one, while in the AOUP the Gaussian clusters are compressed in the transversal direction with respect to the channel as if the wall affects the *x*-variance of the particle distribution inside the wall-clusters. This narrow stripe of clusters slides along the walls changing direction with an average rate ~1/*D*_*r*_ = *τ*, without breaking away from the walls. Clusters, hexagonally arranged, form in the bulk at very small *U*_0_, behaving in the same way as unconfined clusters, with one of the hexagonal directions aligned with the walls, as confirmed by the study of the *x*-density, *ρ*(*x*), in panel g) which reveals separated peaks. This situation changes by increasing *U*_0_. In the bulk, far from the walls, clusters become unstable: they disappear and reform continuously in time as in the unconfined case, but maintain a clear alignment. As shown in Fig. 4c,d, the inner clusters elongate along the *y*-direction until they collapse in vertical stripes. In this case, the study of *ρ*(*x*) cannot capture this dynamical effect: the central peaks are only less pronounced with respect to the lateral ones and overlap. A further increase of *U*_0_ destroys the stripes order in the inner region, creating a fluid-like homogenous bulk-phase, whose structure is confirmed by the study of the density in panel i) which is roughly homogenous until the occurrence of the first stripes. In the proximity of the walls two stripes clearly separated are stable: the one in front of the wall and the one at the interface with the fluid-region, clearly separated by an empty region. In practice, the wall stripe creates an effective extra wall at distance ~±(*L* − *R*), where active particles accumulate.

Finally, when *U*_0_ is very large we recover the usual behavior of active particles in the presence of walls (not shown), i.e. accumulation near walls without any structures in the bulk region. We remark that the suppression of any ordered structure occurs at larger values of *U*_0_ than in the case of the unconfined system. The presence of the walls, which breaks the rotational symmetry, stabilizes the stripe-phase which cannot form in the absence of walls. As in the unconfined case, the drift of the pattern is controlled by the average active force of the whole system. Nevertheless, we observe that the confinement prevents any motion along the transversal direction of the channel.

## Conclusion

In this work, we have studied a system of active particles in the presence of soft-core repulsive interactions, a coarse-grained model for suspensions of activated complex polymers, such as dendrimers or star polymers. We explored how the active force affects the cluster-crystal phase of the system. We discover the existence of traveling cluster crystals, with a speed induced by the active force. The crystal moves coherently in space, for a typical time which depends on the persistence of the active force, maintaining its hexagonal structure. In addition, the cluster-shape is deeply affected by the strength of the active force which determines its size, until producing an unstable region where at first clusters can exchange particles and then destroy and reform continuously in time. Finally, for large enough self-propulsion, the crystal melts. We have checked in specific cases that such a phenomenology is not restricted to our choice of interparticle potential and we expect it to be present in a large class of soft-core interactions. We explore two different modelizations of the active force, both well-known in the literature, exploiting analogies and differences between them. Besides some differences in the particular parameter values at which transitions occur, the only feature which distinguishes the two descriptions is the cluster shape: for some values of the control parameters they display a central hole in the ABP case, but not in the AOUP. We have explained in detail the reasons for this difference.

Finally, we confine the system into an infinitely long channel to explore the dynamics of active soft repulsive particles. The effective long-range effect of the wall clearly appears, deeply influencing the structure of the pattern until to produce a collapse into a stripe-phase aligned to the walls. Such a phenomenon has not a passive counterpart and is entirely due to the active force.

The consideration of more complicated confining geometries, finite-size for the particles, and the role of repulsive and attractive potentials acting at different scales may give rise to interesting behaviors and applications of the interplay of active forces and aggregated phases of particles. This is material to be explored in the close future.

## Supplementary information

Supplementary File

Supplementary File
