# Supplementary material for: A comparative study between two models of active cluster crystals
Source: Sci Rep. 2019 Nov 13;9:16687. doi: 10.1038/s41598-019-52420-1 (PMC6853940; doi:10.1038/s41598-019-52420-1)
Supplement: Supplementary file 2 — Supplementary File [file 41598_2019_52420_MOESM2_ESM.pdf]

Supplementary Information: a comparative study between two models of self-propelled cluster-crystals

Lorenzo Caprini,[1] Emilio Hernández-García,[2] Cristóbal López,[2] and Umberto Marini Bettolo Marconi[3]

[1]*Gran Sasso Science Institute (GSSI), Via. F. Crispi 7, 67100 L'Aquila, Italy.*
[2]*IFISC (CSIC-UIB), Instituto de Física Interdisciplinar y Sistemas Complejos, Campus Universitat de les Illes Balears, E-07122 Palma de Mallorca, Spain.*
[3]*Scuola di Scienze e Tecnologie, Università di Camerino - via Madonna delle Carceri, 62032, Camerino, Italy*

Description of Movie 1

The Movie is realized with $N = 2 \times 10^3$ particles interacting with a GEM-3 potential, given by Eq.(1) of the main text with $\alpha = 3$. The Movie is composed of four panels each realized with an independent simulation with a different set of parameters. Top panels are obtained with the AOUP model, where the self-propulsion evolves with Eq.(4) of the main text, while bottom panels are realized with the ABP active force dynamics, given by Eq.(3) of the main text. The left column and right one are obtained by fixing $D_r = 1$ (or $\tau = 1$) and $D_r = 0.1$ (or $\tau = 10$), respectively. In each panel, we draw the average active force of each cluster with a green vector in the middle of each cluster. Instead, the black arrow in the middle of the box is the average active force of the whole system. The other parameters involved in the simulations are: $U_0 = 2$, $R = 10^{-1}$, $L = 1$, $\gamma = 1$, $\epsilon = 1$, $T = 10^{-4}U_0^2/\gamma D_r$.

The Movie shows that both ABP and AOUP active forces give rise to a stable drifting pattern, whose typical rate of change in drift direction increases with τ (or equivalently with $1/D_r$).

Derivation of Eqs.(12) and (13) of the main text

To derive Eqs.(12) and (13) of the main text, it is convenient to switch from the differential stochastic equation (2) of the main text in the presence of the ABP active force, to the associated Fokker Planck equation for the probability distribution, $\mathcal{P}(\mathbf{x}, \theta)$, which reads:

$$\frac{\partial}{\partial t}\mathcal{P} = \nabla \cdot (\frac{\nabla U}{\gamma} - U_0)\mathcal{P} + \frac{T}{\gamma}\nabla^2\mathcal{P} + D_r\frac{\partial^2}{\partial\theta^2}\mathcal{P}\,, \tag{1}$$

being the external potential U a harmonic trap of the form:

$$U(x, y) = \frac{k}{2}(x^2 + y^2)\,.$$

Because of the radial symmetry of U we change from Cartesian to Polar coordinates $(x, y, \theta) \to (r, \phi, \theta)$, being $r = \sqrt{x^2 + y^2}$ and $\phi = \arctan(y/x)$, in such a way that the Fokker-Planck equation for the probability distribution function $\tilde{\mathcal{P}}(r, \phi, \theta)$ becomes:

$$\frac{\partial}{\partial t}\tilde{\mathcal{P}} = \frac{\partial}{\partial r}\left[\frac{k}{\gamma}r\tilde{\mathcal{P}} - \frac{T}{\gamma r}\tilde{\mathcal{P}} + \frac{T}{\gamma}\frac{\partial}{\partial r}\tilde{\mathcal{P}} - U_0\tilde{\mathcal{P}}\cos(\theta - \phi)\right] + \left[\frac{T}{\gamma r^2}\frac{\partial^2}{\partial\phi^2}\tilde{\mathcal{P}} + \frac{U_0}{r}\tilde{\mathcal{P}} - \frac{U_0}{r}\sin(\theta - \phi)\frac{\partial}{\partial\phi}\tilde{\mathcal{P}}\right] + D_r\frac{\partial^2}{\partial\theta^2}\tilde{\mathcal{P}}\,. \tag{2}$$

Finding an exact solution of such a partial differential equation is not so easy. Assuming that $\theta \sim \phi$, we can approximate $\sin(\phi - \theta) \approx \phi - \theta$ and $\cos(\phi - \theta) \approx 1$, neglecting terms of order $(\phi - \theta)^2$ and higher. This approximation can hold only in the limit $D_r \to 0$. Assuming that the radial flux (i.e. the first square brackets in Eq.(2)) is zero, we can find an approximate stationary solution for the radial component of $\tilde{\mathcal{P}}$, under the assumption that $\tilde{\mathcal{P}}$ is factorized as the product of a radial component, $\rho(r)$, and an angular component, $f(\theta, \phi)$. The vanishing of the first square bracket in Eq. (2) leads to

$$\tilde{\mathcal{P}}(r, \theta, \phi) \approx f(\theta, \phi)\; r\; \exp\left(\frac{\gamma}{T}\left[-\frac{k}{\gamma}\frac{r^2}{2} + U_0 r\right]\right)\,, \tag{3}$$

where $f(\theta, \phi)$ is to be determined. The radial part of this expression is proportional to $\rho(r)$, and thus $p(\mathbf{x})$ (switching to Cartesian components) is a Gaussian with variance T/k centered around a ring of radius $r^* = \gamma U_0/k$.

Since circular symmetry of the clusters implies translational invariance of the angular components, it is straighforward to derive that $f(\theta,\phi) = g(\phi-\theta)$. Approximating $r \approx r^*$ in the remaining terms of Eq. (2) and using that $\sin(\phi-\theta) \approx \phi-\theta$, we find

$$g(\phi-\theta) \approx \mathcal{N} \exp\left(-\frac{\gamma U_0 r^*}{T+\gamma D_r (r^*)^2} \frac{[\phi-\theta]^2}{2}\right), \tag{4}$$

with $\mathcal{N}$ a normalization factor. Eq.(4) shows that ϕ is distributed as a Gaussian centered at θ, whose variance is

$$\mathrm{Var}(\phi-\theta) = \frac{T+\gamma D_r (r^*)^2}{\gamma U_0 r^*},$$

and demonstrates Eq.(13) of the main text. Combining both results we get an approximate solution of the Fokker-Planck equation, whose validity is restricted to the limit $D_r \to 0$.
